# Psychometric aspects of the Tilburg Pregnancy Distress Scale: data from the HAPPY study

**DOI:** 10.1007/s00737-019-00974-4

**Published:** 2019-05-02

**Authors:** Myrthe G. B. M. Boekhorst, Annemerle Beerthuizen, Maarten Van Son, Veerle Bergink, Victor J. M. Pop

**Affiliations:** 1 Department of Medical and Clinical Psychology, Tilburg University, Warandelaan 2, PO Box 90153, 5000, 5037 AB LE Tilburg, The Netherlands; 2 Department of Psychiatry, Erasmus MC, University Medical Centre Rotterdam, Rotterdam, The Netherlands; 3 Department of Obstetrics and Gynecology, Máxima Medical Centre, Veldhoven, The Netherlands; 4 Department of Clinical and Health Psychology, Utrecht University, Utrecht, The Netherlands; 5 Departments of Psychiatry and Obstetrics, Icahn School of Medicine at Mount Sinai, New York, USA

**Keywords:** Maternal distress, Negative affect, Pregnant women, Delivery, Test-retest reliability, Validity

## Abstract

We previously developed the Tilburg Pregnancy Distress Scale (TPDS). The aim of the current study was to further assess its test-retest reliability, internal consistency, and construct and concurrent validity in 1739 pregnant women. TPDS scores during pregnancy were highly inter-correlated (*r* ≥ .70), with similar findings for its Negative Affect and Partner Involvement subscales. Pregnancy and delivery worries varied in different subgroups of women regarding their obstetric history. Nullipara reported more pregnancy- and delivery-related worries at all trimesters of pregnancy. Women with previous pregnancy-related complications reported more pregnancy-related worries, and those with previous delivery-related problems reported more delivery-related worries than women without these problems in the past. The TPDS seems to be a valid and reliable instrument to assess pregnancy-specific distress.

## Introduction

We previously developed the Tilburg Pregnancy Distress Scale (TPDS; Pop et al. 2011), a short screening scale to measure pregnancy-specific distress, with an emphasis on the mothers own perspective. This 16-item scale has a two-factor structure, highlighting symptoms of *negative affect* (NA, 11 items) and perceived *partner involvement* (PI, 5 items) during pregnancy. The 11-item negative affect scale consists of three subscales: worries about pregnancy (3 items), worries about delivery (5 items), and worries about the postpartum period (3 items). Since its development, the TPDS has been translated into English, Portuguese, Turkish, Spanish, and Bahasa Indonesia (e.g., Ҫapik & Pasinlioglu 2015).

Morrell et al. (2013) emphasized the fact that the TPDS was developed using focus-group interviews as a starting point, and a recent systematic review of self-report questionnaires of anxiety during pregnancy evaluated the TPDS as excellent in terms of its internal consistency and structural validity (Evans et al. 2015). However, it was also concluded that data on hypothesis testing and construct validity was insufficient and data on reliability were not reported (Evans et al. 2015). To address these issues, we designed a study to evaluate test-retest reliability and internal consistency of the TPDS and its subscales. We used repeated measurements of the TPDS in a large sample of pregnant women at three trimesters. Validation analysis was additionally carried out in the current study by investigating the pregnancy and delivery NA subscales in different subgroups of women. In particular, we analyzed possible differences in nulliparous and multiparous women with or without a history of pregnancy/delivery complications and/or spontaneous abortion. It is hypothesized that these subgroups of women will vary in levels of pregnancy and delivery worries during the course of their pregnancy.

## Materials and methods

The design of this large cohort study, the HAPPY (Holistic Approach to Pregnancy and the first Postpartum Year) study, is published elsewhere (Truijens et al. 2014). In total, 2275 women completed online questionnaires at 12, 22, and 32 weeks of pregnancy. Data on important demographic (age) and obstetric characteristics (parity) were missing in 144 women. Of the remaining 2131 women, 188 women (8.8%) failed to return fully completed TPDS questionnaires at all trimesters due to various reasons. Because of this low number, we did not perform a multiple imputation procedure. Especially for test-retest reliability, we considered a rigorous timeframe for the completion of the questionnaires by the remaining 1947 women: a 4-week timeframe of the specified trimester during the time of assessment (12 ± 4 weeks, 22 ± 4 weeks, and 32 ± 4 weeks). In total, 1755 women met these rigorous criteria. Considering the PI subscale items, women without a partner (*n* = 12) were excluded from the analysis. A final sample of 1739 women were suitable for analysis, and of whom the characteristics (demographics, lifestyle habits, obstetrics, and psychiatric history) were not different from the original sample as published elsewhere in the current journal (Truijens et al. 2017), in which scores of the Edinburgh (Postnatal) Depression Scale (E(P)DS; Cox et al. 1987) were also repeatedly assessed at all trimesters. The study was approved by the Psychology Ethics Committee of Tilburg University (protocol number EC-2012.25). Written informed consent was obtained from all individual participants included in the study.

Information about obstetric characteristics was collected at 12 weeks of pregnancy. Pregnancy-specific distress (maternal distress) was measured using the 16-item TPDS (Pop et al. 2011). The 4-point Likert scale results in total scores ranging from 0 to 48, with higher scores indicating greater levels of distress. The total subscale score of NA (11 items) ranges from 0 to 33 and PI (5 items) from 0 to 15, with higher scores representing greater levels of negative affect and poorer partner involvement, respectively.

Statistical analyses were performed using the Statistical Package for Social Sciences (SPSS version 22.0, IBM, Chicago, Illinois, USA). At all trimesters, an Exploratory Factor Analysis (EFA) was executed on the 16 items of the TPDS using principal components analysis (PCA) with oblimin rotation using a 2-factor solution as previously described (Pop et al. 2011). Confirmative factor analysis (CFA) was used to test the stability of the factor structures. The comparative fit index (CFI), the Tucker-Lewis Index (TLI), the normed fit index (NFI), and the root mean square error of approximation (RMSEA) were used to evaluate model fit. A CFI of ≥ 0.80 in combination with a NFI of ≥ 0.80 and a RMSEA of < 0.06 are generally considered as indicators of adequate fit of a model (Browne and Cudeck 1993). To determine the internal consistency of the scale, the Cronbach’s alpha coefficient of the TPDS and its subscales were calculated at all trimesters. Test-retest reliability was measured by calculating the correlations between the total TPDS scores and the total subscale (NA and PI) scores of the same participants at three different trimesters (Pearson *r*, two-tailed). There was a three-month time period between assessments, the suggested minimum time gap for test-retest reliability assessment (Kline 2000). Construct validity was assessed using hypotheses testing by comparing mean scores of the NA subscales in various subgroups of women throughout gestation using *t* test and one-way repeated measures analysis of variance (ANOVA). Concurrent validity was assessed by calculating the correlations between the TPDS and E(P)DS scores at all trimesters (Pearson *r,* two-tailed).

## Results

### Factor analyses

EFA performed at 12, 22, and 32 weeks confirmed a similar 2-factor structure as in the original study: a 5-item PI dimension and 11-item NA dimension with 3 subscales: worries about pregnancy, worries about delivery, and worries about the postpartum period (Pop et al. 2011). CFA showed an adequate model fit at all trimesters with the original structure. The figures of the CFI, NFI, TLI, and RMSEA were 0.92, 0.93, 0.94, and 0.06 at 12 weeks, 0.94, 0.95, 0.95, and 0.05 at 22 weeks, 0.95, 0.96, 0.96, and 0.04 at 32 weeks of pregnancy, respectively.

### Reliability analyses

The Cronbach’s alpha of the total TPDS at 12, 22, and 32 weeks was 0.74, 0.76, and 0.75, respectively; of the 11-item NA subscale, these values were 0.77, 0.78, and 0.77, respectively; of the 5-item subscale PI, these figures were 0.74, 0.79, and 0.81 respectively. Table 1 shows that the total TPDS scores during pregnancy are highly inter-correlated (*r* ≥ .70, appropriate test-retest reliability), with similar findings for its subscales.

**Table 1:** Correlation matrix of the TPDS scores at successive assessments to measure test-retest reliability, mean scores, and ranges (*n* = 1739)

	TPDS 12 wks	NA 12 wks	PI 12 wks	TPDS 22 wks	NA 22 wks	PI 22 wks	TPDS 32 wks	NA 32 wks	PI 32 wks	Mean (SD)	Range
TPDS 12 wks	1	.87**	.58**	.75**	.68**	.40**	.70**	.63**	.39**	10.7 (5.3)	0–38
NA 12 wks	–	1	.10**	.65**	.76**	.08*	.59**	.69**	.07*	6.49 (4.3)	0–31
PI 12 wks	–	–	1	.44**	.12**	.68**	.43**	.13**	.65**	4.22 (2.6)	0–14
TPDS 22 wks	–	–	–	1	.86**	.61**	.79**	.68**	.48**	10.3 (5.4)	0–34
NA 22 wks	–	–	–	–	1	.12**	.68**	.77*	.11**	6.01 (4.3)	0–28
PI 22 wks	–	–	–	–	–	1	.48**	.13**	.76**	4.33 (2.8)	0–14
TPDS 32 wks	–	–	–	–	–	–	1	.87**	.60**	10.9 (5.6)	0–36
NA 32 wks	–	–	–	–	–	–	–	1	.12**	6.47 (4.5)	0–30
PI 32 wks	–	–	–	–	–	–	–	–	1	4.47 (2.8)	0–14

*p < .01;**p < .001 (two-tailed)

*wks*, weeks; *TPDS*, Tilburg Pregnancy Distress Scale; *NA*, negative affect; *PI*, partner involvement, *SD*; standard deviation

### Validity analyses/hypothesis testing

**Fig. 1:**
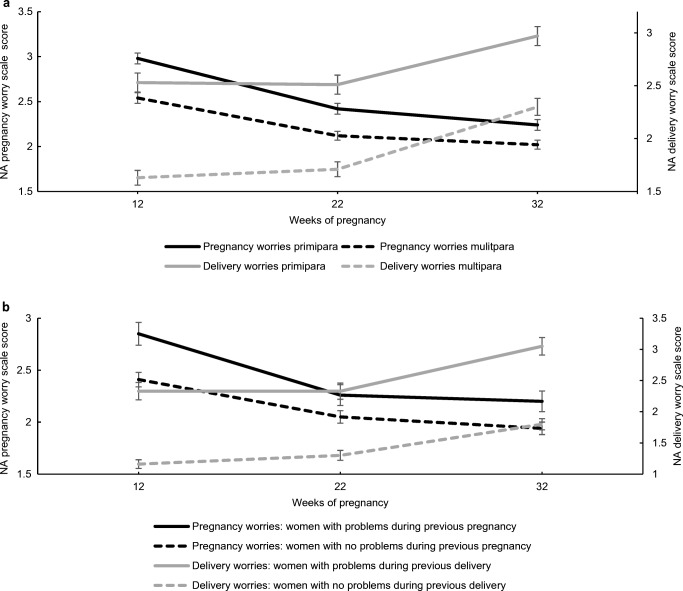
Mean negative affect scores in different groups of women during the course of pregnancy **a** change in mean scores on the pregnancy and delivery worry scale during the course of pregnancy for primiparous and multiparous women, **b** change in mean scores during the course of pregnancy regarding pregnancy-related worries for multipara with and without problems during a previous pregnancy, and regarding delivery-related worries for multipara with and without problems during a previous delivery. NA; negative affect

In total, 870 women were nulliparous and 869 multiparous. As shown in Fig. 1a, nullipara reported significantly higher mean scores on the pregnancy worry scale compared to multipara at 12 (M nullipara = 2.98, M multipara = 2.54; *p* < .001), 22 (M nullipara = 2.42, M multipara = 2.12; *p* < .001), and 32 (M nullipara = 2.24, M multipara = 2.02; *p* = .003) weeks of pregnancy. Furthermore, results showed that pregnancy-related worry scores significantly decreased during the course of pregnancy in the study population as a whole (*F* (1.89, 3275.8) = 183.8, *p* < .001). Post hoc analysis showed that mean pregnancy-related worry scores decreased significantly from 12 to 22 weeks of pregnancy (M = 2.76 and M = 2.27, respectively, *p* < .001), as well as from 22 to 32 weeks of pregnancy (M = 2.27 and M = 2.13, respectively, *p* < .001).

Figure 1 a also shows that nullipara had significantly higher delivery worry scores compared with multipara at 12 (M nullipara = 2.53, M multipara = 1.63; *p* < .001), 22 (M nullipara = 2.51, M multipara = 1.72; *p* < .001), and 32 (M nullipara = 2.97, M multipara = 2.30; *p* < .001) weeks of pregnancy. Results also showed a significant increase in delivery worry scores over time in the study population as a whole: *F* (1.93, 3356.6) = 91.3, *p* < 0.001. Post hoc analysis showed that mean delivery-related worry scores did not increase from 12 to 22 weeks of pregnancy (M = 2.08 and M = 2.11, respectively, *p* = 1.0), but did significantly increase from 22 to 32 weeks of pregnancy (M = 2.11 and M = 2.63, respectively, *p* < .001).

In total, 460 (26%) women reported a previous spontaneous abortion. These women reported significantly more pregnancy-related worries at 12 weeks of gestation (M = 3.01, *p* < .001) but not at the two other trimesters (data not shown). Of the 869 multipara, there were 261 (30%) women who reported problems in a previous pregnancy. Figure 1 b shows that they had significantly higher pregnancy worry scores compared with women without these problems at 12 (M = 2.85 and M = 2.41, respectively, *p* < .001) and 32 (M = 1.94 and M = 2.20, respectively, *p* = .028) weeks of pregnancy. In all multipara, the scores decreased towards the end of gestation: *F* (1.86, 1612.3) = 66.2, *p* < .001. Post hoc analysis showed that mean pregnancy-related worry scores decreased significantly from 12 to 22 weeks of pregnancy (M = 2.54 and M = 2.12, respectively, *p* < .001), but not from 22 to 32 weeks of pregnancy (M = 2.12 and M = 2.02, respectively, *p* = .051).

Of the 869 multipara, 349 (40%) reported problems during a previous delivery. Figure 1 b shows that they had significantly higher delivery worry scores compared with women without these problems during a previous delivery at 12 (M = 2.33 and M = 1.16, respectively, *p* < .001), 22 (M = 2.33 and M = 1.30, respectively, *p* < .001), and 32 (M = 3.05 and M = 1.80, respectively, *p* < .001) weeks of pregnancy. The scores in all multipara increased towards the end of pregnancy: *F* (1.92, 1668.6) = 72.5, *p* < .001. Post hoc analysis showed that mean delivery-related worry scores did not increase from 12 to 22 weeks of pregnancy (M = 1.63 and M = 1.72, respectively, *p* = .365), but did significantly increase from 22 to 32 weeks of pregnancy (M = 1.72 and M = 2.30, respectively, *p* < .001).

### Concurrent validity

At 12, 22, and 32 weeks of pregnancy the E(P)DS correlated significantly with the TPDS *r* = 0.50, *r* = 0.53, and *r* = 0.54, respectively (*p* < 0.001, two-tailed).

## Discussion

The current study showed satisfactory test-retest coefficients for the TPDS and its subscales at all trimesters of pregnancy, as well as adequate internal consistency. We found that (i) pregnancy worry scores decreased towards end term; (ii) nulliparous women report higher pregnancy worry symptom scores than multiparous women; (iii) nullipara with a spontaneous abortion in history scored higher on the pregnancy worry scale, but this effect disappeared as pregnancy progressed; (iv) delivery worry scores increased towards the end of term in all women, with higher scores in nullipara than in multipara throughout pregnancy; (v) multipara with problems during a previous pregnancy had a higher pregnancy worry score in the first and third trimester in comparison to multipara without these problems; and (vi) multipara with problems during a previous delivery had a higher delivery worry scores throughout pregnancy than multipara without these problems.

It is likely that nullipara reports more pregnancy-related worries than multipara because they have not experienced a normal evolving pregnancy. Other studies have also found that nullipara report more pregnancy-related anxiety throughout pregnancy compared with multipara (Blackmore et al. 2016; Huizink et al. 2016). Moreover, these scores are likely to decrease as pregnancy progresses, especially after 20 weeks of gestation if the standardized ultrasound confirms a normal pregnancy. Women predominantly expect reassurance about their baby’s health after an ultrasound (Öhman & Waldenström 2008), and that women’s total worries and anxiety tend to decrease after a routine ultrasound (Ekelin et al. 2009). Also, women who have previously experienced a spontaneous abortion will report more pregnancy-related worries during the first 16 weeks, reflecting the overall high-risk period for abortion. Overall, these findings suggest that these TPDS NA subscales indeed measure what they intend to measure: worries specific to pregnancy and delivery, respectively. Furthermore, the fact that the E(P)DS correlated significantly with the TPDS and its subscales but with limited R^2^ figures (25–29%) suggest that the TPDS assesses concepts which are different from depression, although related.

A strength of the current study is the large sample size. Another strength is the longitudinal design, which allowed us to measure distress between different subgroups of women at three trimesters of pregnancy. A limitation is that the current study population includes more highly educated and Caucasian women than in the general Dutch population (Statistics the Netherlands, 2019). Generalizability to the general population may therefore be limited. This could be explained by the fact that the current study was based in the south-east of the Netherlands, a more highly educated population compared with the general Dutch population. This region was named “smartest area of the world” in 2011 by the international think-tank Intelligent Community Forum (ICF) in New York. Pregnancy-specific distress instruments, such as the TPDS, the Cambridge Worry Scale (Green et al. 2003), and the Prenatal Distress Questionnaire (Yali & Lobel 1999), were recently reviewed, concluding that further psychometric evaluation of existing pregnancy-specific scales was needed (Evans et al. 2015).

In conclusion, the current study provides substantial evidence that the TPDS and its subscales contain good psychometric properties with regard to test-retest reliability, internal consistency, construct validity, and concurrent validity.
